# Immunomodulatory drugs have divergent effects on humoral and cellular immune responses to SARS-CoV-2 vaccination in people living with rheumatoid arthritis

**DOI:** 10.1038/s41598-023-50263-5

**Published:** 2023-12-21

**Authors:** Jenna M. Benoit, Jessica A. Breznik, Jann C. Ang, Hina Bhakta, Angela Huynh, Braeden Cowbrough, Barbara Baker, Lauren Heessels, Sumiya Lodhi, Elizabeth Yan, Joycelyne Ewusie, Ishac Nazy, Jonathan Bramson, Matthew S. Miller, Sasha Bernatsky, Maggie J. Larché, Dawn M. E. Bowdish, the SUCCEED Investigator Group

**Affiliations:** 1 Department of Medicine, McMaster University, Hamilton, ON Canada; 2 McMaster Immunology Research Centre, Hamilton, ON Canada; 3 Firestone Institute of Respiratory Health, St. Joseph’s Healthcare, Hamilton, ON Canada; 4 McMaster Institute for Research on Aging, McMaster University, Hamilton, ON Canada; 5 Department of Biochemistry, McMaster University, Hamilton, ON Canada; 6 Department of Medicine, University of Ottawa, Ottawa, ON Canada; 7 Department of Health Research Methods, Evidence, and Impact (HEI), McMaster University, Hamilton, ON Canada; 8 Department of Medicine, McGill University, Montreal, QC Canada

**Keywords:** Autoimmunity, RNA vaccines, Immunosuppression

## Abstract

Understanding the efficacy of SARS-CoV-2 vaccination in people on immunosuppressive drugs, including those with rheumatoid arthritis (RA), is critical for their protection. Vaccine induced protection requires antibodies, CD4^+^ T cells, and CD8^+^ T cells, but it is unclear if these are equally affected by immunomodulatory drugs. Here, we determined how humoral and cellular SARS-CoV-2 vaccination responses differed between people with RA and controls, and which drug classes impacted these responses. Blood was collected from participants with RA on immunomodulatory drugs and controls after their second, third, and fourth SARS-CoV-2 vaccinations. Receptor binding domain (RBD)-specific antibodies were quantified by ELISA. Spike-specific memory T cells were quantitated using flow cytometry. Linear mixed models assessed the impact of age, sex, and immunomodulatory drug classes on SARS-CoV-2 vaccination responses. Compared to non-RA controls (n = 35), participants with RA on immunomodulatory drugs (n = 62) had lower anti-RBD IgG and spike-specific CD4^+^ T cell levels, but no deficits in spike-specific CD8^+^ T cells, following SARS-CoV-2 vaccination. Use of costimulation inhibitors was associated with lower humoral responses. JAK inhibitors were associated with fewer spike-specific CD4^+^ T cells. Participants with RA on immunomodulatory drugs mounted weaker responses to SARS-CoV-2 vaccination, with different drug classes impacting the cellular and humoral compartments.

## Introduction

Immunosuppressive medications such as steroids, disease modifying anti-rheumatic drugs (DMARDs), and biologics are commonly prescribed to treat autoimmune disorders including rheumatoid arthritis (RA). DMARDs, which include methotrexate, broadly suppress inflammatory responses, whereas biologics target and block specific inflammatory mediators or pathways (e.g. interleukin-6 and tumor necrosis factor inhibitors)^1^. Multiple large-scale studies have reported an increased risk of SARS-CoV-2 infection, hospitalization, and death in people with rheumatic diseases such as RA^2,3^. Furthermore, certain immunomodulatory drug classes, such as TNF and costimulation (i.e. Cytotoxic T-lymphocyte Antigen 4, CTLA-4 Ig) inhibitors, are associated with weaker humoral responses to SARS-CoV-2 vaccination^4–6^.

Antibodies targeting the spike protein of SARS-CoV-2, and the neutralization capacity of these antibodies, have been widely explored as potential correlates of protection following SARS-CoV-2 vaccination^7,8^. Vaccine-elicited neutralizing antibodies were protective against symptomatic infection with the ancestral strain of SARS-CoV-2, and maintained efficacy against earlier variants of concern^7,8^. SARS-CoV-2 variants of concern, such as omicron variants, have the ability to evade humoral immunity due to the accumulation of mutations, particularly in the spike protein^9^. While antibodies are often used as a marker of protection following SARS-CoV-2 vaccination, spike-specific T cells are also critical for vaccine induced protection^10,11^. Omicron variants in particular have accumulated mutations in the spike protein that contribute to their ability to evade humoral immune responses generated by vaccination against the ancestral strain, while T cell responses are largely conserved^9,12,13^. Thus, when assessing SARS-CoV-2 vaccination responses it is necessary to evaluate both cellular and humoral immunity.

Given the likely role of T cells in long-term and cross variant protection, it is important to explore if different immunomodulatory drug classes impact T cell responses compared with humoral responses, or even differentially impact CD4^+^ vs CD8^+^ responses. Furthermore, the question of whether different immunomodulatory drug classes affect the subpopulations (Th1, Th2, Th17, T regulatory) of spike-specific CD4^+^ T cells could offer insight into how protective their responses will be in the context of SARS-CoV-2 infection. In order to better understand the impact of different drug classes on humoral and cellular responses to SARS-CoV-2 vaccination, we explored both arms of immunity after 2, 3, and 4 doses of SARS-CoV-2 vaccines in people with RA, who are on immunomodulatory drugs. We found that while costimulation inhibitors are associated with weaker humoral responses in terms of antibody levels, JAK inhibitors are associated with fewer spike-specific CD4^+^ but not CD8^+^ T cells. Participants on JAK inhibitors also displayed an altered spike-specific CD4^+^ T cell skew, with a greater proportion of T regulatory (Treg) cells. This study therefore highlights that different drug classes may affect both the development and the functional skew of humoral and cellular responses to SARS-CoV-2 vaccination.

## Results

### Participant and control demographics

Altogether 62 participants on immunomodulatory drugs for RA (median age 63.0 years [interquartile range (IQR) 55.0–68.0]; 84% female sex), and 35 control participants (median age 64.0 [IQR 54.0–70.3]; 66% female sex) who did not have autoimmune conditions and were not on immunomodulatory drugs, provided samples at multiple timepoints surrounding their second, third, and fourth SARS-CoV-2 vaccinations (Table 1). Age did not differ significantly between groups, but the RA group was much more predominantly female than controls. Most participants with RA were on DMARDs (n = 49, 79%), 40% (n = 25) were on TNF inhibitors, 17% (n = 11) were on JAK inhibitors, and 19% (n = 12) were on costimulation inhibitors. A small number (n = 12, 19%) were on oral steroids. Many participants were on more than one class of immunomodulatory drug. Participants with RA had a shorter interval between their second and third SARS-CoV-2 vaccinations, and third and fourth SARS-CoV-2 vaccinations, than controls (Table 1).

**Table 1:** Participant demographics and immunomodulatory drugs.

	Rheumatoid arthritis	Controls	P value
Total participants	62	35	N/A
Age, median years (IQR)	63.0 (55.0–68.0)	64.0 (54.0–70.3)	ns^a^
Sex, % female subjects (n)	84 (52)	66 (23)	0.045^b^
First dose vaccine type	48 BNT162b2 5 mRNA-1273 9 ChAdOx1	23 BNT162b2 4 mRNA-1273 7 ChAdOx1	ns^b^
Second dose vaccine type	44 BNT162b2 14 mRNA-1273 4 ChAdOx1	27 BNT162b2 6 mRNA-1273 1 ChAdOx1	ns^b^
Third dose vaccine type	39 BNT162b2 12 mRNA-1273	18 BNT162b2 12 mRNA-1273	ns^b^
Fourth dose vaccine type	18 BNT162b2 23 mRNA-1273 1 bivalent	6 BNT162b2 6 mRNA-1273 2 bivalent	ns^b^
Days between dose 1 and dose 2 (median ± SD)	71.0 ± 20.2	72.0 ± 20.6	ns^a^
Days between dose 2 and dose 3 (median ± SD)	152.5 ± 33.3	183.0 ± 27.5	< 0.0001^a^
Days between dose 3 and dose 4 (median ± SD)	119.0 ± 48.5	203.0 ± 67.5	0.0004^a^
Steroids^c^, % (n)	19 (12)	N/A	N/A
DMARDs^d^, % (n)	79 (49)	N/A	N/A
TNF (tumor necrosis factor) and TNF receptor inhibitors^e^, % (n)	40 (25)	N/A	N/A
Janus Kinase Inhibitors, % (n)	17 (11)	N/A	N/A
Costimulation inhibitor (Abatacept), % (n)	19 (12)	N/A	N/A
SARS-CoV-2 Infections, % (n)	24 (15)	14 (5)	ns^b^

Vaccine type information is unknown for one control participant, and the fourth dose for one participant with RA. Vaccination history reported for all participants who provided samples after that dose.

*SD* standard deviation, *DMARDs* disease modifying anti-rheumatic drugs.

^a^Student’s *t* test.

^b^Fisher’s exact test.

^c^Prednisone and methylprednisolone.

^d^Sulfasalazine, leflunomide, methotrexate, mycophenolate mofetil, and hydroxychloroquine.

^e^Includes adalimumab, etanercept, infliximab, and golimumab.

### Participants with RA exhibit lower antibody responses to SARS-CoV-2 vaccination

**Figure 1:**
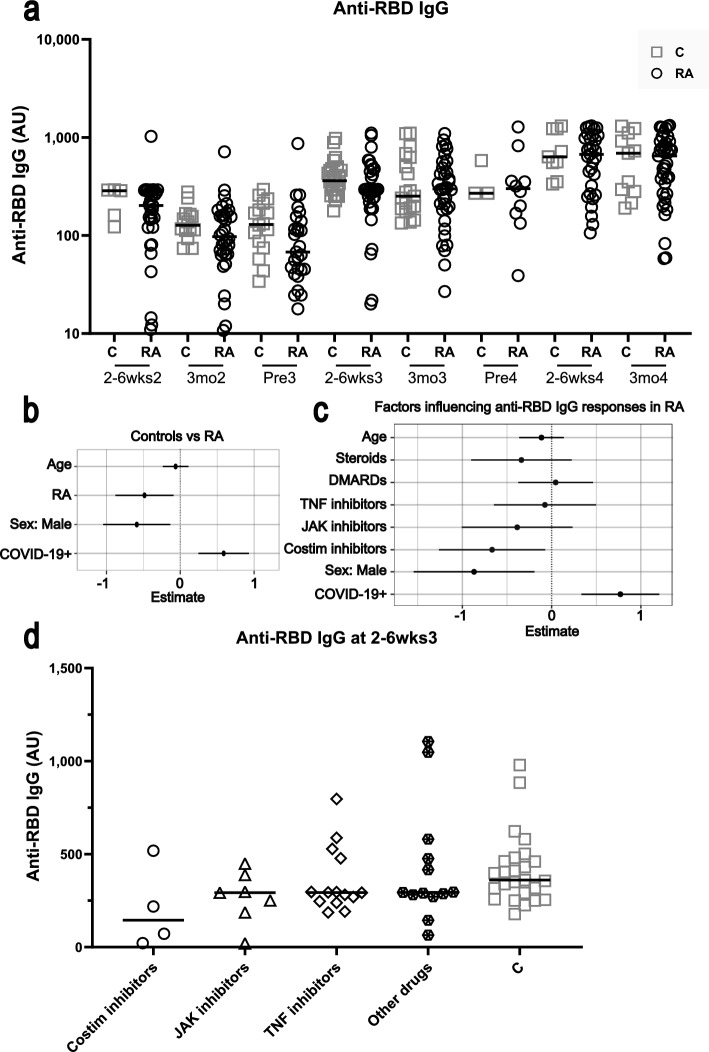
Serum anti-RBD IgG in participants with RA and controls following SARS-CoV-2 vaccination. (**a**) Anti-RBD IgG in the serum was measured by ELISA for participants with RA (open circles), and controls (C, grey squares). The solid line represents the median. PreX is pre dose X, 2–6wksX is 2–6 weeks post dose X, 3moX is 3 months post dose X. (**b**) Multivariable linear mixed model estimates (Log-twofold changes ± SD) comparing RA and C cohorts, and the effects of different parameters on anti-RBD IgG levels. (**c**) Multivariable linear mixed model estimates (Log-2-fold changes ± SD) within the RA cohort of the effects of different parameters, including immunomodulatory drugs, on anti-RBD IgG levels following SARS-CoV-2 vaccination. Costim denotes costimulation. (**d**) Anti-RBD IgG levels in participants with RA on costimulation inhibitors, JAK inhibitors, TNF inhibitors, other drug classes (steroids ± DMARDs), and controls (C, grey squares) at 2–6 weeks post dose 3.

**Figure 2:**
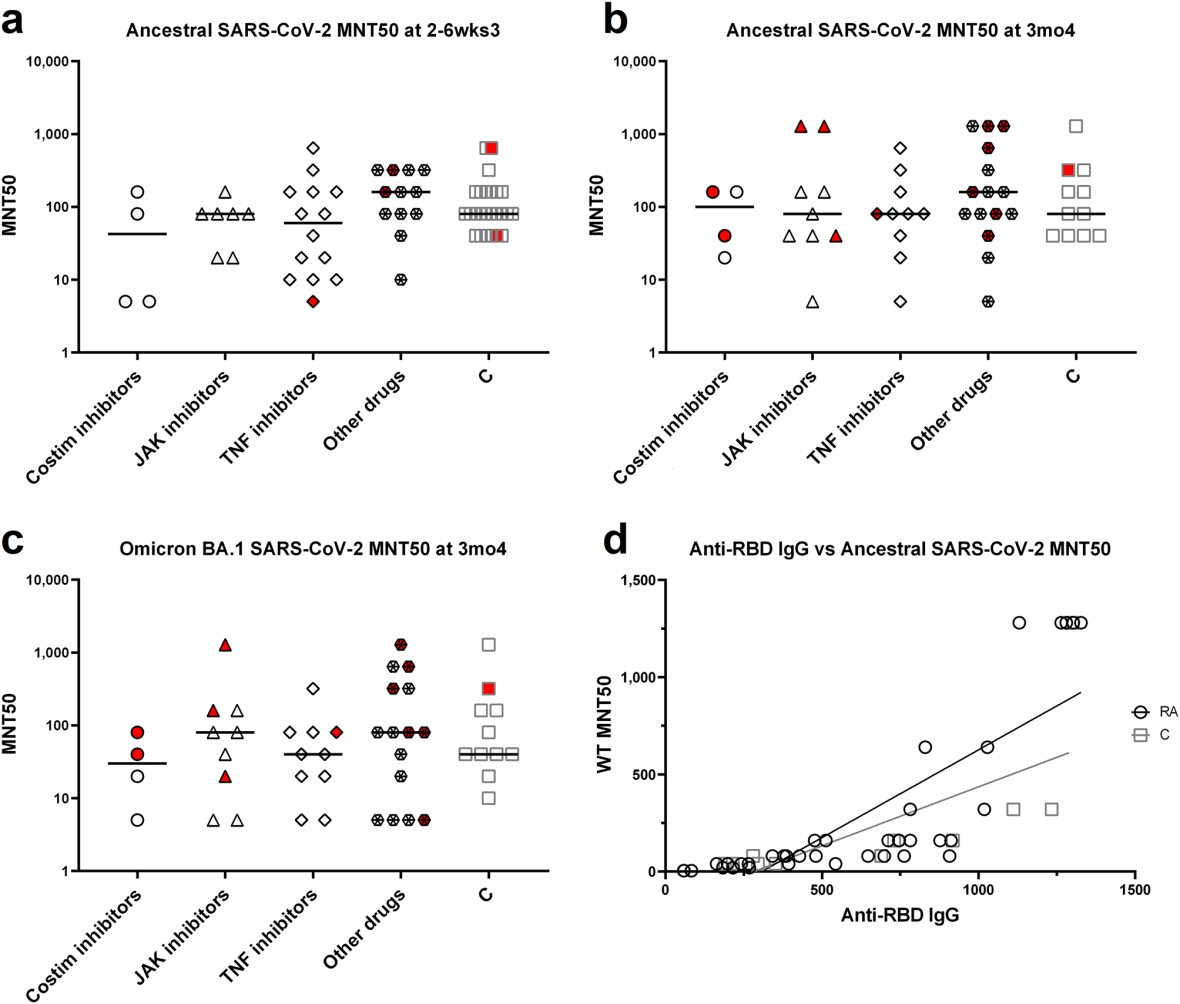
Neutralization capacity against ancestral and omicron BA.1 SARS-CoV-2 in participants with RA and controls. MNT50 against ancestral SARS-CoV-2 in participants with RA on costimulation inhibitors, JAK inhibitors, TNF inhibitors, other drug classes (steroids ± DMARDs), and controls (C, grey squares) at 2–6 weeks post dose 3 (**a**), and 3 months post dose 4 (**b**). (**c**) MNT50 against the omicron BA.1 variant of SARS-CoV-2 in participants with RA and controls at 3 months post dose 4. Comparisons were made by Brown-Forsythe tests with Dunnett’s T3 post-hoc tests. (**d**) Relationship between anti-RBD IgG levels and ancestral (WT) MNT50 in participants with RA and controls assessed using simple linear regression and the Pearson correlation coefficient (R = 0.81 for RA, R = 0.71 for C). Symbols filled in red indicate participants who have previously had a SARS-CoV-2 infection.

Anti-receptor binding domain (RBD) antibodies are associated with neutralization and early viral control of SARS-CoV-2^14^. Using a multivariable linear mixed model with timepoint as a time-varying covariate, we found that participants living with RA had lower levels of anti-RBD IgG overall than controls following SARS-CoV-2 vaccination (Fig. 1a,b, Table 2). Unlike other statistical tests, the multivariable linear mixed does not identify specific timepoints where responses differ between RA and control cohorts, but rather determines whether the controls and RA groups are different when all other factors including time are accounted for. While age did not clearly associate with antibody levels, males had lower levels of anti-RBD IgG than females when looking in both the RA and control cohorts (Fig. 1b, Table 2). As expected, participants with a previous SARS-CoV-2 infection (COVID-19 +) had higher levels of serum anti-RBD IgG (Fig. 1b). Compared with anti-RBD IgG levels at 2–6 weeks post dose 2, antibody levels were significantly increased following dose 3 and dose 4 in controls and participants with RA (Table 2, Supplemental Fig. 1a,b). Since the participants with RA had significantly weaker humoral responses to SARS-CoV-2 vaccination, with notable variation within the cohort, we next examined which drug classes contributed to this deficit. Using a multivariable linear mixed model, which looked only within the RA cohort, it was found that individuals with RA on costimulation inhibitors had lower anti-RBD IgG responses to vaccination than those taking other classes of drugs (Fig. 1c,d, Table 2). While the model suggests that costimulation inhibitors may have a detrimental impact on anti-RBD IgG levels following SARS-CoV-2 vaccination, the limited sample size was not sufficiently powered to definitively determine drug impacts, highlighting the need for larger scale studies to validate these findings. To provide further insight into the impact costimulation inhibitors have on anti-RBD IgG levels, we broke down the participants with RA into different drug groups at 2–6 weeks post dose 3 (the timepoint with the most samples) and graphed their antibody levels (Fig. 1d). Note only four participants were on costimulation inhibitors at this timepoint, as drug regimens changed throughout the study and different participants provided samples at different timepoints. The heterogeneity in drug regimens among participants with RA, and the changes in these regimens throughout the study, do however reflect the reality of treating autoimmune disorders. Interestingly, at 2–6 weeks post dose 3 and 3 months after dose 4, no drug class impacted the neutralization capacity of the antibodies in participants with RA, against either the ancestral SARS-CoV-2 or the omicron BA.1 variant (Fig. 2a–c). This is in accordance with our finding that anti-RBD IgG levels only moderately correlated with ancestral SARS-CoV-2 neutralization at 3 months post dose 4 (Fig. 2d).

**Table 2:** Multivariable linear mixed modelling of the impact of variables on humoral and cellular responses following COVID-19 vaccination.

Variable	Effect estimate (± SD)	Parameter	P value^a^
Rheumatoid arthritis	− 0.48 ± 0.20	Anti-RBD IgG	< 0.05
	− 1.07 ± 0.34	Spike-specific CD4^+b^	< 0.05
	0.14 ± 0.41	Spike-specific CD8^+^	0.74
Age	− 0.06 ± 0.09	Anti-RBD IgG	0.47
	− 0.09 ± 0.15	Spike-specific CD4^+^	0.54
	− 0.22 ± 0.18	Spike-specific CD8^+^	0.23
Male sex	− 0.59 ± 0.23	Anti-RBD IgG	0.01
	− 0.46 ± 0.39	Spike-specific CD4^+^	0.25
	0.92 ± 0.48	Spike-specific CD8^+^	0.06
COVID-19 infection (positive)	0.58 ± 0.17	Anti-RBD IgG	< 0.001
	0.49 ± 0.30	Spike-specific CD4^+^	0.10
	− 0.001 ± 0.35	Spike-specific CD8^+^	0.99
Timepoint^c^ (compared with 2–6 weeks post dose 2)			
3-months post dose 2	− 0.75 ± 0.16	Anti-RBD IgG	< 0.001
	− 0.52 ± 0.27	Spike-specific CD4^+^	0.06
	− 0.22 ± 0.30	Spike-specific CD8^+^	0.47
Pre-dose 3	− 0.88 ± 0.18	Anti-RBD IgG	< 0.001
	− 0.62 ± 0.30	Spike-specific CD4^+^	< 0.05
	− 0.07 ± 0.33	Spike-specific CD8^+^	0.83
2–6 weeks post dose 3	0.93 ± 0.16	Anti-RBD IgG	< 0.001
	0.47 ± 0.27	Spike-specific CD4^+^	0.09
	0.61 ± 0.31	Spike-specific CD8^+^	< 0.05
3-months post dose 3	0.69 ± 0.17	Anti-RBD IgG	< 0.001
	0.32 ± 0.28	Spike-specific CD4^+^	0.25
	0.96 ± 0.32	Spike-specific CD8^+^	< 0.05
Pre dose 4	0.79 ± 0.26	Anti-RBD IgG	< 0.05
	0.59 ± 0.43	Spike-specific CD4^+^	0.17
	1.04 ± 0.49	Spike-specific CD8^+^	< 0.05
2–6 weeks post dose 4	1.88 ± 0.19	Anti-RBD IgG	< 0.001
	0.57 ± 0.32	Spike-specific CD4^+^	0.07
	1.67 ± 0.36	Spike-specific CD8^+^	< 0.001
3-months post dose 4	1.51 ± 0.18	Anti-RBD IgG	< 0.001
	0.59 ± 0.31	Spike-specific CD4^+^	0.05
	1.30 ± 0.34	Spike-specific CD8^+^	< 0.001
Immunomodulatory drugs			
Steroids	− 0.34 ± 0.29	Anti-RBD IgG	0.24
	0.04 ± 0.43	Spike-specific CD4^+^	0.93
	− 0.83 ± 0.50	Spike-specific CD8^+^	0.09
DMARDs	0.04 ± 0.21	Anti-RBD IgG	0.83
	− 0.58 ± 0.33	Spike-specific CD4^+^	0.08
	− 0.31 ± 0.37	Spike-specific CD8^+^	0.40
TNF inhibitors	− 0.08 ± 0.29	Anti-RBD IgG	0.80
	0.41 ± 0.43	Spike-specific CD4^+^	0.34
	0.48 ± 0.50	Spike-specific CD8^+^	0.34
JAK inhibitors	− 0.39 ± 0.32	Anti-RBD IgG	0.23
	− 2.57 ± 0.48	Spike-specific CD4^+^	< 0.001
	0.05 ± 0.56	Spike-specific CD8^+^	0.93
Costimulation inhibitor	− 0.67 ± 0.30	Anti-RBD IgG	< 0.05
	− 0.60 ± 0.47	Spike-specific CD4^+^	0.21
	− 0.14 ± 0.53	Spike-specific CD8^+^	0.80

^a^Significance cut-off α = 0.05.

^b^Spike-specific CD4^+^ and CD8^+^ refer to T cells.

^c^Estimates from mixed model including both controls and participants with RA.

### Participants with RA have lower spike-specific CD4^+^ T cells than controls following SARS-CoV-2 vaccination

**Figure 3:**
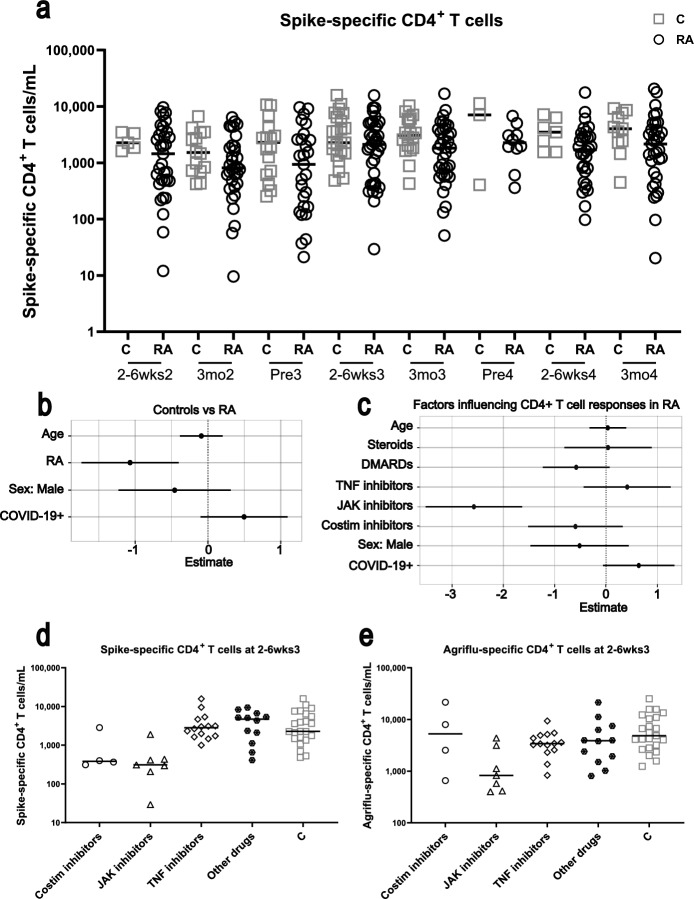
Levels of spike-specific CD4^+^ T cells in participants with RA on immunomodulatory drugs, and controls. (**a**) The numbers of spike-specific CD4^+^ T cells were measured using AIM assays in participants with RA (open circles), and controls (C, grey squares). The solid line represents the median. PreX is pre dose X, 2–6wksX is 2–6 weeks post dose X, 3moX is 3 months post dose X. (**b**) Multivariable linear mixed model estimates (Log-twofold changes ± SD) comparing RA and C cohorts, and the effects of different parameters on spike-specific CD4^+^ T cell numbers. (**c**) Multivariable linear mixed model estimates (Log-twofold changes ± SD) within the RA cohort of the effects of different parameters, including immunomodulatory drugs, on spike-specific CD4^+^ T cells following SARS-CoV-2 vaccination. (**d**) Spike-specific CD4^+^ T cell levels in participants with RA on costimulation inhibitors, JAK inhibitors, TNF inhibitors, other drug classes (steroids ± DMARDs), and controls (C, grey squares) at 2–6 weeks post dose 3. (**e**) Influenza-specific (Agriflu-specific) CD4^+^ T cell levels in participants with RA on different immunomodulatory drug classes at 2–6 weeks post third SARS-CoV-2 vaccination. Comparisons were made by Brown-Forsythe tests with Dunnett’s T3 post-hoc tests.

Spike-specific T cells are also thought to be critical for vaccine induced protection^10,11,15^. Participants with RA had lower levels of spike-specific CD4^+^ T cells than controls (Fig. 3a,b, Table 2). Age, sex, and previous SARS-CoV-2 infection did not significantly influence the levels of spike-specific CD4^+^ T cells in either participants with RA or controls (Fig. 3b, Table 2). Levels of spike-specific CD4^+^ T cells were also not increased after the third and fourth vaccinations, compared with post dose 2, in either controls or participants with RA (Table 2, Supplemental Fig. 1c,d). Given the lower numbers of spike-specific CD4^+^ T cells in participants with RA, a multivariable linear mixed model was used to investigate which drugs were associated with this deficit. The use of JAK inhibitors in the RA drug regimens was associated with lower spike-specific CD4^+^ T cells (Fig. 3c,d, Table 2). We visualized this in Fig. 3d using the 2–6 weeks post dose 3 timepoint. At 2–6 weeks post dose 3, participants on costimulation inhibitors trended towards lower levels of spike-specific CD4^+^ T cells, however by 3 months post dose 4 this gap had closed (Supplemental Fig. 2a). A trend towards lower antigen-specific CD4^+^ T cell levels in participants treated with JAK inhibitors was also seen when we measured the influenza-specific (Agriflu) CD4^+^ T cell levels at the 2–6 week post dose 3 timepoint (Fig. 3e). JAK inhibitors did not however clearly alter the CD4^+^ T cell response to polyclonal TCR specificity-independent stimulation using Cytostim (Supplemental Fig. 2b).

### Spike-specific CD4^+^ T cell skew may differ by drug class

**Figure 4:**
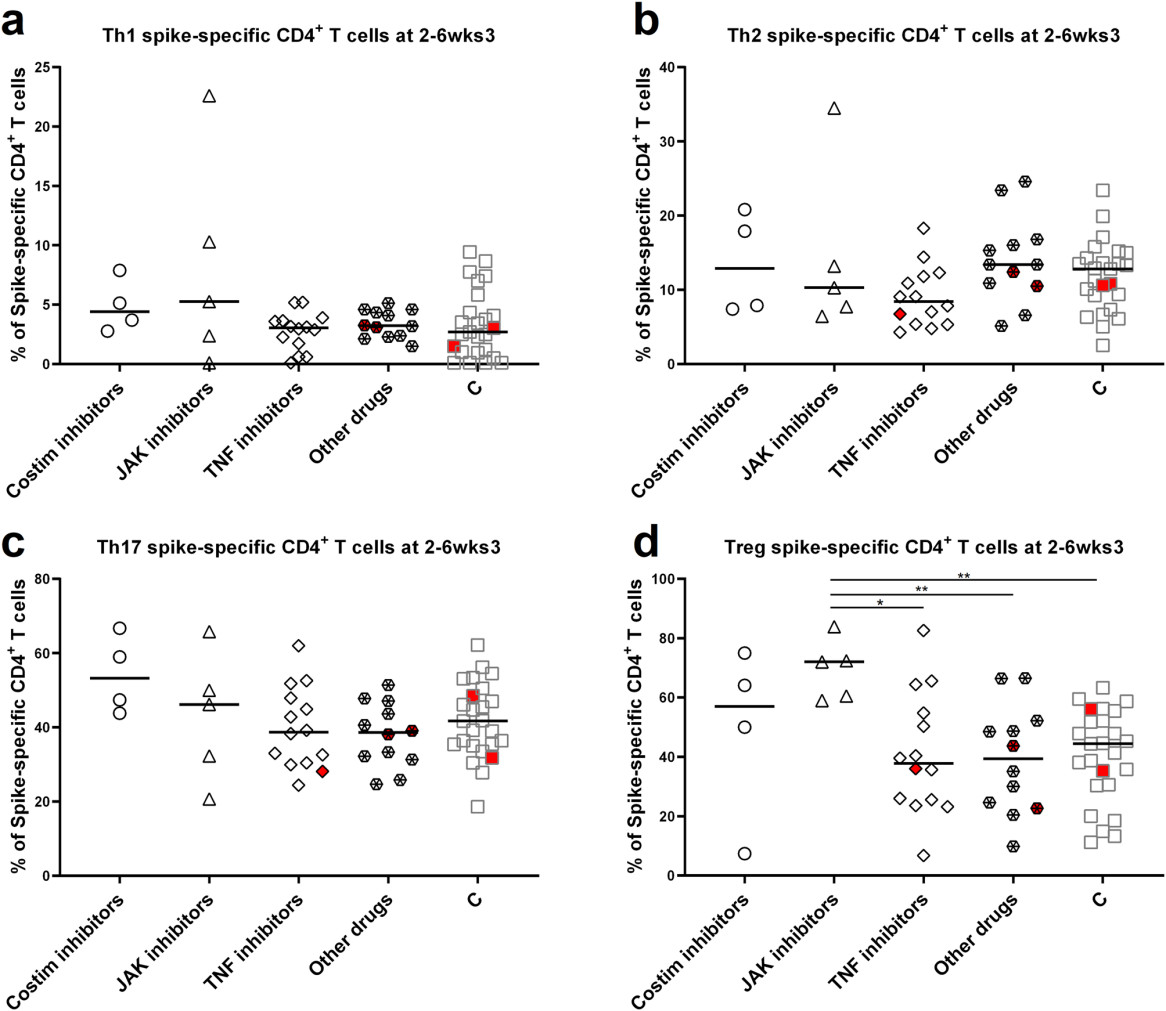
Skew of spike-specific CD4^+^ T cells in participants with RA, on immunomodulatory drugs, and controls at 2–6 weeks post dose 3. (**a**) The frequency of spike-specific CD4^+^ T cells, determined by AIM assays, displaying a Th1 skew (CXCR3^+^CCR6^−^CCR4^−^) in participants with RA and controls (C, grey squares). Participants with RA were broken down by drug class into those taking costimulation inhibitors, JAK inhibitors, TNF inhibitors, or other drug classes (steroids ± DMARDs). Participants were only plotted if there were > 20 CD4^+^AIM^+^ events, allowing accurate determination of phenotype. (**b**) The frequency of spike-specific CD4^+^ T cells displaying a Th2 skew (CXCR3^−^CCR6^−^CCR4^+^) in participants with RA and controls. (**c**) The frequency of spike-specific CD4^+^ T cells displaying a Th17 skew (CXCR3^−^CCR6^+^CCR4^+^) in participants with RA and controls. (**d**) The frequency of spike-specific CD4^+^ T cells displaying a T regulatory phenotype (Tregs, CD25^+^CD39^+^) in participants with RA and controls. Symbols filled in red indicate participants who have previously had a SARS-CoV-2 infection. Comparisons were made by Brown-Forsythe tests with Dunnett’s T3 post-hoc tests. p < 0.05 *, p < 0.01 **.

Considering that spike-specific CD4^+^ T cell levels were lower in participants with RA on JAK inhibitors, we also wondered if the phenotypic skew of these cells differed depending on drug classes. When looking 2–6 weeks after dose 3, we found that the frequencies of spike-specific Th1 (CXCR3^+^CCR6^−^CCR4^−^), Th2 (CXCR3^−^CCR6^−^CCR4^+^), and Th17 (CXCR3^+^CCR6^+^CCR4^+^) CD4^+^ T cells did not differ between those on different RA drug treatments, or controls (Fig. 4a–c). Surprisingly, the frequency of spike-specific T regulatory cells (Tregs, CD25^+^CD39^+^) was significantly higher in participants with RA on JAK inhibitors than in those on TNF inhibitors, other drugs, or controls (Fig. 4d). In contrast, participants with RA on JAK inhibitors had lower spike-specific CD4^+^ Th2 and Th17 cell numbers (cells per mL of whole blood) than controls but did not display higher numbers of spike-specific Tregs (Supplemental Fig. 3a–d). This suggests that the increased frequency of spike-specific Tregs in this population is not due to an increase in the numbers of these cells, but rather due to the lower levels of the other CD4^+^ subpopulations. The influence of this change in spike-specific CD4^+^ T cell skew on vaccine-induced protection against SARS-CoV-2 remains to be determined.

### Participants with RA and controls have similar numbers of spike-specific CD8^+^ T cells following SARS-CoV-2 vaccination

**Figure 5:**
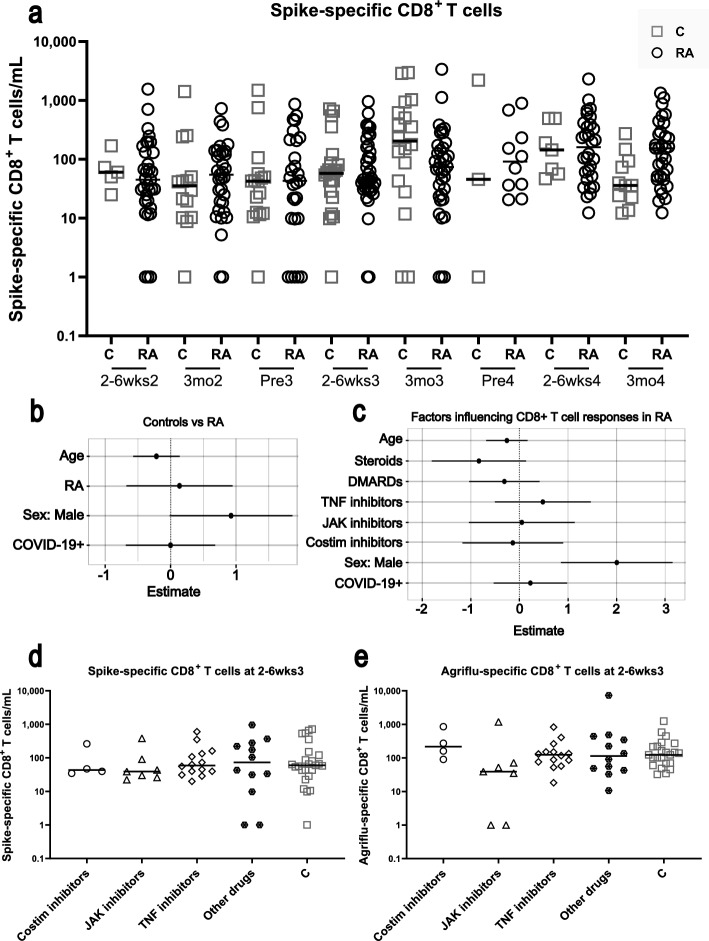
Levels of spike-specific CD8^+^ T cells in participants with RA on immunomodulatory drugs, and controls. (**a**) The number of spike-specific CD8^+^ T cells were measured using AIM assays in participants with RA (open circles), and controls (C, grey squares). The solid line represents the median of a group. PreX is pre dose X, 2-6wksX is 2–6 weeks post dose X, 3moX is 3 months post dose X. (**b**) Multivariable linear mixed model estimates (Log-twofold changes ± SD) comparing RA and C cohorts, and the effects of different parameters on spike-specific CD8^+^ T cell numbers. (**c**) Multivariable linear mixed model estimates (Log-twofold changes ± SD) within the RA cohort of the effects of different parameters, including immunomodulatory drugs, on spike-specific CD8^+^ T cells following SARS-CoV-2 vaccination. (**d**) Spike-specific CD8^+^ T cell levels in participants with RA on costimulation inhibitors, JAK inhibitors, TNF inhibitors, other drug classes (steroids ± DMARDs), and controls (C, grey squares) at 2–6 weeks post dose 3. (**e**) Influenza-specific (Agriflu-specific) CD8^+^ T cell levels in participants with RA on different immunomodulatory drug classes at 2–6 weeks post third SARS-CoV-2 vaccination. Comparisons were made by Brown-Forsythe tests with Dunnett’s T3 post-hoc tests.

In contrast to what was observed with the spike-specific CD4^+^ T cell numbers, participants with RA overall did not exhibit any deficits in numbers of spike-specific CD8^+^ T cells (Fig. 5a,b, Table 2). When looking at both the control and RA samples, spike-specific CD8^+^ T cell numbers increased after the third and fourth SARS-CoV-2 vaccinations, compared with post dose 2 (Table 2, Supplemental Fig. 1e,f). Age and previous SARS-CoV-2 infection did not clearly associate with levels of spike-specific CD8^+^ T cells in either the control or RA samples (Fig. 5b,c). When looking within the RA cohort only, no drug class was significantly associated with spike-specific CD8^+^ T cell numbers (Fig. 5c). Among participants with RA, males did however exhibit more robust spike-specific CD8^+^ T cell responses than females (Fig. 5b,c, Table 2). Finally, to illustrate any differences, or lack thereof, in antigen-specific CD8^+^ T cell responses between drug groups, we plotted the samples from the RA cohort at 2–6 weeks post dose 3 (Fig. 5d,e). The spike- and influenza-specific CD8^+^ T cell responses were not significantly different between drug groups (Fig. 5d,e). There were also no drug class associated deficits in the CD8^+^ T cell response to polyclonal TCR specificity-independent stimulation using Cytostim (Supplemental Fig. 2c).

## Discussion

Understanding the impact of different immunomodulatory drug classes on humoral and cellular responses to SARS-CoV-2 vaccination is critical for the protection of vulnerable populations, including those with RA. In particular, whether certain drug classes affect only cellular or only humoral responses to SARS-CoV-2 vaccination, or affect the skew of the cellular responses, still needs to be fully elucidated. In this study we found that participants on RA treatments had lower anti-RBD IgG levels than non-RA controls following their second, third, and fourth SARS-CoV-2 vaccinations. Additional vaccine doses did boost the antibodies levels, as did previous SARS-CoV-2 infection. Inclusion of costimulation inhibitors in their immunomodulatory drug regimen was associated with weaker humoral responses. This finding is consistent with other studies that showed the costimulation inhibitor abatacept weakened humoral responses to pneumococcal and SARS-CoV-2 vaccination^6,16,17^. Interestingly, we were unable to establish if any RA drug significantly affected neutralization capacity of the antibodies against either the ancestral or omicron BA.1 SARS-CoV-2. The primary limitation of our study is the small overall sample size, with low numbers of participants on certain drug classes, which we addressed by using the minimum number of clinically relevant covariates. Age and sex are known to impact immune responses, and we were able to account for this impact on humoral and cellular responses to SARS-CoV-2 vaccination^18–20^. Our study highlights the need for studies with larger sample sizes in the future to detect differential drug treatment effects on vaccination responses with a higher degree of certainty.

Though antibody levels are commonly examined following vaccination, spike-specific T cells are also thought to be critical for vaccine induced protection, and protection against variants of concern^10,11,15,21^. We found that participants on RA treatments had lower numbers of spike-specific CD4^+^ T cells following SARS-CoV-2 vaccination. Use of JAK inhibitors was associated with this significantly weaker response^22^. Other studies have reported decreased IFN-γ responses upon stimulation with the spike protein in participants with immune-mediated inflammatory disorders on DMARDs, costimulation inhibitors, and TNF inhibitors^22,23^. These studies looked at earlier timepoints, following the first and second SARS-CoV-2 vaccinations, highlighting that booster vaccinations may be critical for participants on these drugs to mount responses comparable to those seen in controls. We saw this in our own study, as participants on costimulation inhibitors appeared to have lower spike-specific CD4^+^ T cell levels at 2–6 weeks post dose 3, but these levels had become comparable to controls following dose 4.

JAK inhibitors not only impacted the number of spike-specific CD4^+^ T cells, but also the phenotypic subpopulations. While the frequencies of Th1, Th2, and Th17 cells were similar to other drug classes, the frequency of Tregs was increased in participants with RA on JAK inhibitors. This was not due to an increase in the number of Tregs, but likely due to the decrease in the number of Th17 and Th2 cells in this group, allowing Tregs to account for more of the remaining spike-specific CD4^+^ T cell population. To our knowledge, the impact of JAK inhibitors on the skew of CD4^+^ T cell responses following SARS-CoV-2 vaccination has not been previously reported. However, JAK inhibitors have been reported to alter T cell skews in graft-versus-host disease^24^. The shift of the spike-specific CD4^+^ T cell population towards a regulatory phenotype may decrease the viral clearance of SARS-CoV-2 in these participants upon infection, further dampening their vaccine-induced protection^25,26^. The impact of JAK inhibitors on SARS-CoV-2 infection remains to be definitively determined, with conflicting reports in the literature^27,28^. In contrast, previous studies have shown that the presence of high numbers of CD8^+^ T cells correlates with improved SARS-CoV-2 infection survival in patients with hematologic cancer, and in non-human primate models^10,29^. We observed that participants with RA on various classes of immunomodulatory drugs mounted spike-specific CD8^+^ T cell responses that were comparable to those found in non-RA controls, similar to what has been previously reported in participants with RA and controls after the third SARS-CoV-2 vaccination^22^.

In summary, our study determined that participants with RA on immunomodulatory drugs generally mount weaker humoral and CD4^+^ T cell responses to SARS-CoV-2 vaccination than controls. However, the drug classes which impact these arms of immunity differ. Costimulation inhibitors were associated with weaker humoral responses, while JAK inhibitors were associated with lower levels of spike-specific CD4^+^ T cells. Moving forward, it will be critical to consider which drug classes patients are taking, as the drugs could differentially impact infection risk and long-term cross variant protection.

## Methods

### Participant recruitment and study design

From May 2021 to February 2023, adult (18+) participants classified as having RA (n = 62) according to the criteria from the European League Against Rheumatism (EULAR)/American College of Rheumatology (ACR) were recruited from a tertiary care rheumatology clinic in Hamilton, Ontario. Adult (18+) controls C, (n = 35) without autoimmune disease and not on immunomodulatory drugs, were recruited in the local community. Exclusion criteria (for both RA and controls) were significant immunodeficiency syndromes such as HIV, any prior/current treatment with rituximab, acute febrile illness on the scheduled blood collection date, pregnancy, anyone involved in another new vaccine clinical trial, and anyone undergoing chemotherapy. The age distribution of the RA and control groups were similar.

### Sample collection

Participants had their blood drawn at up to 8 timepoints: 2–6 weeks (RA n = 33, C n = 5) and 3-months after their second vaccination (RA n = 34, C n = 14), before dose 3 (RA n = 27, C n = 16), 2–6 weeks post dose 3 (RA n = 40, C n = 25), 3-months post dose 3 (RA n = 38, C n = 19), before dose 4 (RA n = 10, C n = 3), 2–6 weeks post dose 4 (RA n = 31, C n = 9), and 3 months post dose 4 (RA n = 39, C n = 11). Study design was cross-sectional based on sample availability, in that not all participants provided samples for all timepoints. Table 1 reports demographic information for all enrolled participants.

Venous blood was collected in sodium heparin vacutainer tubes. One non-heparinized vacutainer tube was also collected and centrifuged to separate the serum as per manufacturer directions (Becton Dickinson). Serum was stored at − 80 °C until use.

### ELISAs

Serum was thawed and anti-SARS-CoV-2 receptor binding domain (RBD) immunoglobulins were evaluated by ELISA as previously described, with serum diluted within the linear range^30,31^. SARS-CoV-2 infections were determined by a positive PCR or rapid test, or by seroconversion using an anti-nucleocapsid IgG ELISA^32^.

### Neutralization assays

Serum was thawed and antibody neutralization capacity assessed using live ancestral and omicron BA.1 SARS-CoV-2 cell culture assays as previously described^33^. Results reported as MNT50 (geometric microneutralization titers at 50%).

### Activation induced marker assays

To quantitate SARS-CoV-2 memory CD4^+^ and CD8^+^ T cells, activation induced marker assays were used as previously described^30^. Briefly, peptides derived from the SARS-CoV-2 spike protein (SARS-CoV-2 S Immunodominant Peptivator, Miltenyi Biotec, 1 µg/ml), influenza peptides (AgriFlu, Afluria Tetra inactivated influenza vaccine 2020–2021 season, Seqirus, 4 µl of 0.12 µg/µl), or Cytostim (Miltenyi Biotec, 0.5 µl/well) were used to stimulate 100 µl of whole blood for 48 h at 37 °C. IMDM was added to each well to bring the final volume to 200 µl.

Characterization of T cell populations were then performed using fluorochrome-conjugated antibodies against CD3 (1:50, clone UCHT1, BD Biosciences), CD4 (1:50, clone SK3, BD Biosciences), CD25 (1:50, clone M-A251, BD Biosciences), CD134 (1:100, clone ACT-35, BioLegend), CD39 (1:25, clone A1, BioLegend), CD8 (1:50, clone RPA-T8, BD Biosciences), CD137 (1:50, clone 4B4-1, BioLegend), CD69 (clone FN50, BioLegend), CXCR3 (1:25, clone G025H7, BioLegend), CCR6 (1:25, clone G034E3, BioLegend), and CCR4 (1:50, clone L291H4, BioLegend) suspended in Brilliant Stain Buffer Plus (BD Biosciences) and phosphate buffered saline (PBS). Samples were fixed using 1xFix/Lyse solution (eBioscience), washed with PBS, and resuspended in FACS Wash (0.5% (w/v) bovine serum albumin (BSA), 5 mM EDTA (pH 7.4–7.6) in PBS). AIM^+^CD8^+^ and AIM^+^CD4^+^ T cells that responded to the presented peptides (antigen-specific) and thus expressed activation markers (i.e., CD69^+^CD137^+^ for CD8; CD25^+^CD134^+^ for CD4) were quantitated by flow cytometry. We refer to the cells as spike-specific due to their activated signature in response to exposure to the spike protein. CD4^+^ T cell subsets were identified by chemokine receptor expression: Th1 (CXCR3^+^CCR6^−^CCR4^−^), Th2 (CXCR3^−^CCR4^+^CCR6^−^), and Th17 (CXCR3^−^CCR4^+^CCR6^+^)^32^. Tregs were identified as CD4+ CD39+^34^. Cell numbers were quantitated using CountBright Absolute Counting Beads (Invitrogen).

The flow cytometry gating strategy can be seen in Supplemental Fig. 4. Samples were acquired on a Cytoflex LX (4 laser, Beckman Coulter) using CytExpert software. Data analysis was conducted with FlowJo version 10 (TreeStar).

### Statistics

Data were analyzed in R version 4.1.2 (R Core Team 2022) using the tidyverse, lmerTest, and emmeans packages. Antibody and T cell data were log transformed and then analyzed using multivariable linear mixed models. The first objective was to determine if there were differences in the antibody or T cell responses to SARS-CoV-2 vaccination between participants with RA and controls. To address objective 1, the first mixed models compared the humoral and cellular responses between participants with RA and controls, not considering drug classes. The second objective was to determine which drugs may impact the humoral or cellular responses to SARS-CoV-2 vaccination in participants with RA, with the associated mixed model looking only within the RA cohort. Given our primary limitation being sample size, we selected the minimum number of appropriate, clinically relevant covariates for the mixed models (age, sex, vaccination types, any prior COVID-19 infection). Changes in drug regimens of participants throughout the study were accounted for in the model, and assigned based on whether or not a patient was on that drug at the timepoint. Fisher’s exact tests and Student’s *t* tests used to compare group demographics. In GraphPad version 8, Brown-Forsythe tests (one-way ANOVAs accounting for unequal standard deviations) with Dunnett’s T3 post-hoc tests were used to assess differences in T cell responses to influenza peptides and polyclonal stimulation (cytostim), as these measures were collected at one timepoint. P < 0.05 was considered statistically significant.

### Study approval

Study recruitment and procedures were reviewed and approved by the Hamilton Integrated Research Ethics Board (HiREB) protocol #13307. Participants provided informed consent for sample and data collection and publication prior to participation. All procedures were performed in accordance with the approved study protocol.

### Supplementary Information

Supplementary Information 1.

Supplementary Information 2.

## Data Availability

Data and statistical code for analyses are available upon request to Dawn Bowdish.
