# Supplementary material for: Immunomodulatory drugs have divergent effects on humoral and cellular immune responses to SARS-CoV-2 vaccination in people living with rheumatoid arthritis
Source: Sci Rep. 2023 Dec 21;13:22846. doi: 10.1038/s41598-023-50263-5 (PMC10739702; doi:10.1038/s41598-023-50263-5)
Supplement: Supplementary file 2 — Supplementary Information 2. [file 41598_2023_50263_MOESM2_ESM.pdf]

Immunomodulatory drugs have divergent effects on humoral and cellular immune responses to SARS-CoV-2 vaccination in people living with rheumatoid arthritis

Jenna M. Benoit[1,2,3], Jessica A. Breznik[1,2,4], Jann C. Ang[5], Hina Bhakta[1], Angela Huynh[1], Braeden Cowbrough[1,2], Barbara Baker[1], Lauren Heessels[1], Sumiya Lodhi[6], Elizabeth Yan[1], Joycelyne Ewusie[7], Ishac Nazy[1], Jonathan Bramson[1,2], Matthew S. Miller[2,5], Sasha Bernatsky[8], Maggie J. Larché[1,2], Dawn ME Bowdish[1,2,3]* and the SUCCEED investigator group**.

Affiliations

[1]Department of Medicine, McMaster University, Hamilton, Ontario, Canada.

[2]McMaster Immunology Research Centre, Hamilton, Ontario, Canada.

[3]Firestone Institute of Respiratory Health, St. Joseph's Healthcare, Hamilton, Ontario, Canada.

[4]McMaster Institute for Research on Aging, McMaster University, Hamilton, Ontario, Canada.

[5]Department of Biochemistry, McMaster University, Hamilton, Ontario, Canada.

[6]Department of Medicine, University of Ottawa, Ottawa, Ontario, Canada

[7]Department of Health Research Methods, Evidence, and Impact (HEI), McMaster University, Hamilton, Ontario, Canada.

[8]Department of Medicine, McGill University, Montreal, Quebec, Canada.

* A list of authors and their affiliations appears at the end of the paper

Supplemental Materials

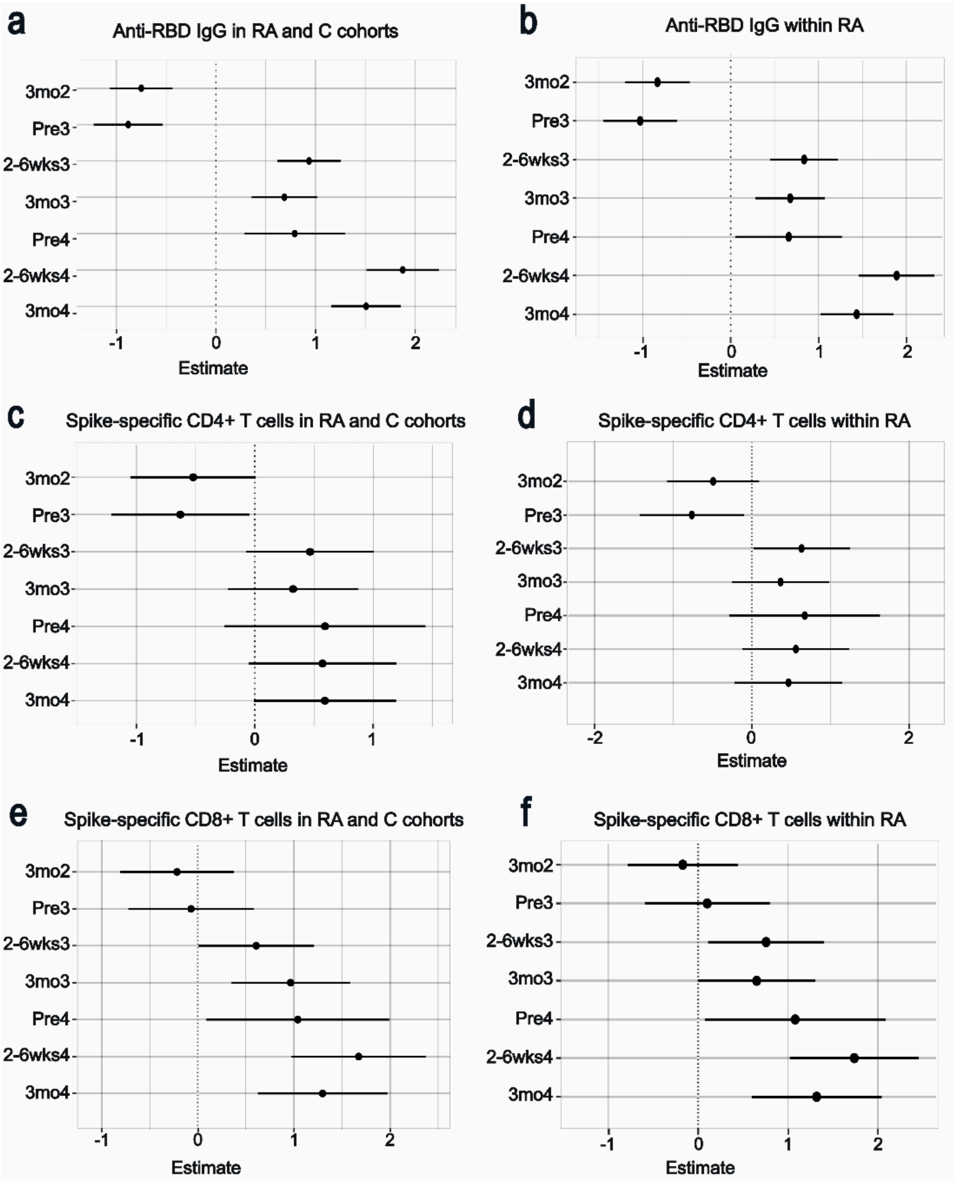

Supplemental Figure 1. Impact of timepoint and dose on SARS-CoV-2 vaccination responses. (a) Multivariable linear mixed model estimates (Log-2 fold changes) examining RA and C cohorts, and the impact of timepoint on anti-RBD IgG levels. (b) Multivariable linear mixed model estimates (Log-2 fold changes) examining the impact of timepoint and vaccine doses on anti-RBD IgG levels within the RA cohort. (c, d) Multivariable linear mixed model estimates (Log-2 fold changes) examining the impact of timepoint and vaccine doses on spike-specific $CD4^+$ T cell levels in RA and C cohorts (c), and within the RA cohort only (d). (e, f) Multivariable linear mixed model estimates (Log-2 fold changes) examining the impact of timepoint and vaccine doses on spike-specific $CD8^+$ T cell levels in RA and C cohorts (e), and within the RA cohort only (f).

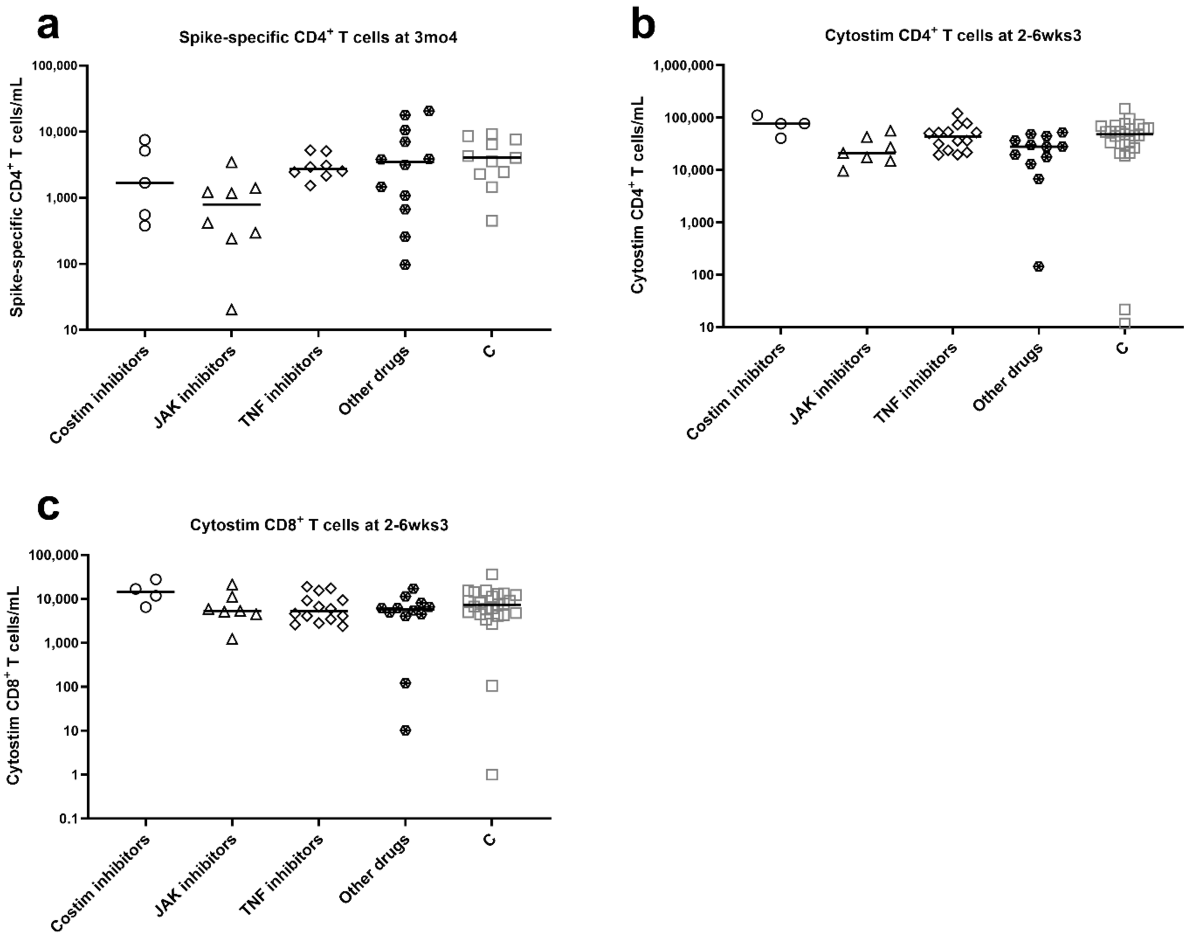

Supplemental Figure 2. CD4+ and CD8+ T cell responses to polyclonal stimulation broken down by drug group. (a) Spike-specific CD4+ T cell levels in participants with RA on costimulation inhibitors, JAK inhibitors, TNF inhibitors, other drug classes (steroids +/- DMARDs), and controls (C, grey squares) at 3 months post dose 4. (b) The number of CD4+ T cells and CD8+ T cells (c) activated in response to Cytostim were measured using AIM assays in participants with RA on costimulation inhibitors, JAK inhibitors, TNF inhibitors, other drug classes (steroids +/- DMARDs), and controls (C, grey squares) at 2-6 weeks post dose 3. The solid line represents the median of a group. Comparisons were made by Brown-Forsythe tests with Dunnett’s T3 post-hoc tests.

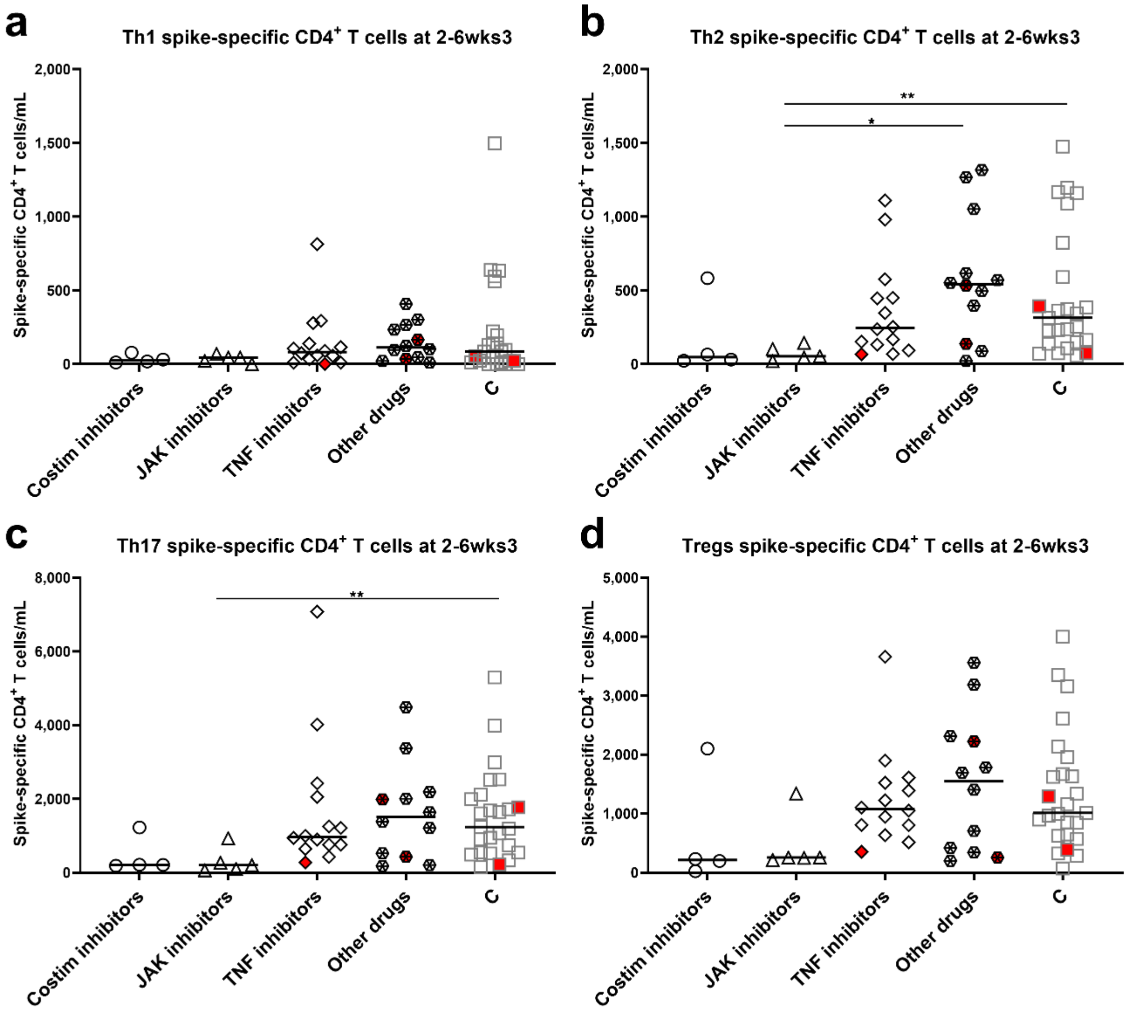

Supplemental Figure 3. Skew of spike-specific CD4+ T cells in participants with RA, on immunomodulatory drugs, and controls at 2-6 weeks post dose 3. (a) The number of spike-specific $CD4^+$ T cells per mL of whole blood, determined by AIM assays, displaying a Th1 skew ($CXCR3^+CCR6^-CCR4^-$) in participants with RA and controls (C, grey squares). Participants with RA were broken down by drug class into those taking costimulation inhibitors, JAK inhibitors, TNF inhibitors, or other drug classes (steroids +/- DMARDs). Participants were only plotted if there were >20 $CD4^+AIM^+$ events, allowing accurate determination of phenotype. (b) The number of spike-specific $CD4^+$ T cells displaying a Th2 skew ($CXCR3^-CCR6^-CCR4^+$) in participants with RA and controls. (c) The number of spike-specific $CD4^+$ T cells displaying a Th17 skew ($CXCR3^-CCR6^+CCR4^+$) in participants with RA and controls. (d) The number of spike-specific $CD4^+$ T cells displaying a T regulatory skew (Tregs, $CD25^+CD39^+$) in participants with RA and controls. Symbols filled in red indicate participants who have previously had a SARS-CoV-2 infection. Comparisons were made by Brown-Forsythe tests with Dunnett's T3 post-hoc tests. $p < 0.05$ *, $p < 0.01$ **.

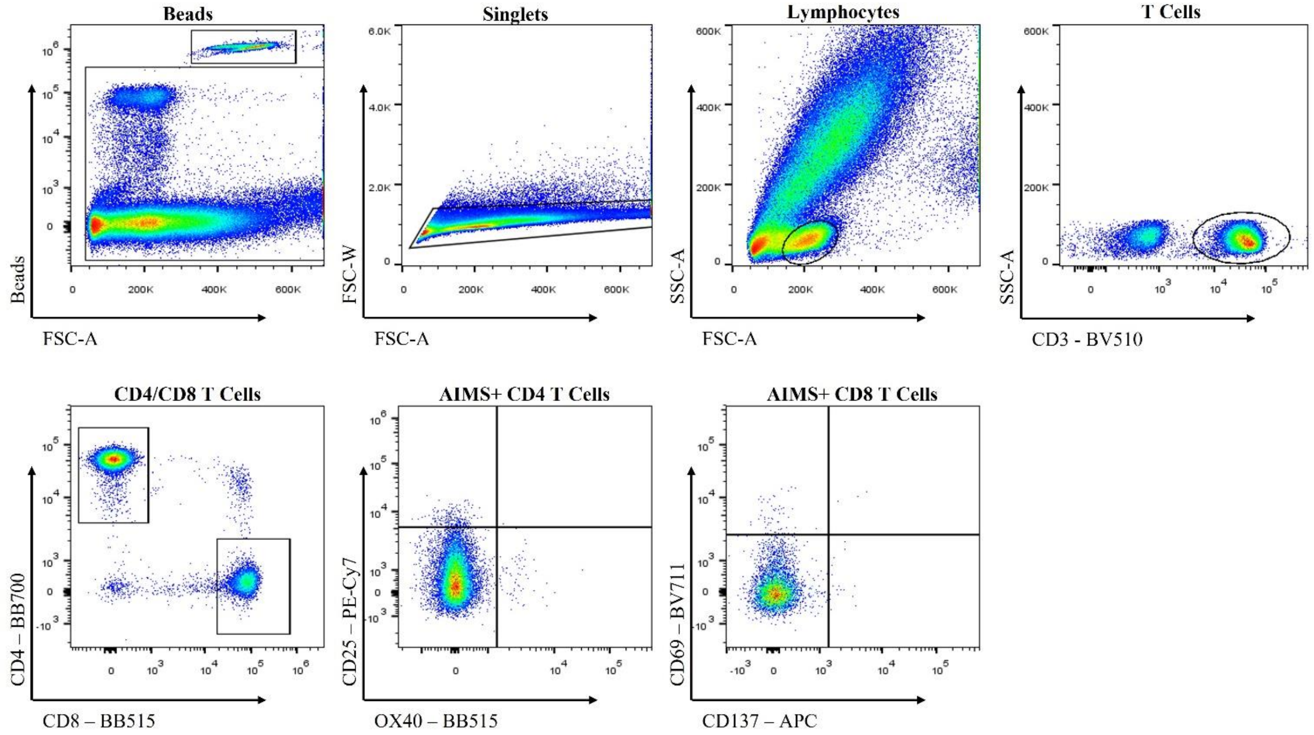

Supplemental Figure 4. AIM assay gating strategy. Count beads were separated from the other events, followed by doublet exclusion and gating on the lymphocyte population. T cells were then identified by expression of CD3 and divided into $CD4^+$ and $CD8^+$ subsets. AIM^+ $CD4^+$ T cells co-expressed CD25 and OX40, while AIM^+ $CD8^+$ T cells co-expressed CD69 and CD137.

Appendix 1. SUCCEED Investigator Group non-author collaborator list.

Vincent Piguet, Department of Medicine, University of Toronto, Toronto, Ontario, Canada.

Stephanie Garner, Department of Medicine, McMaster University, Hamilton, Ontario, Canada.

Hugues Allard-Chamard, Department of Medicine, Université de Sherbrooke, Sherbrooke, Quebec, Canada.

Charles Bernstein, Department of Medicine, University of Manitoba, Winnipeg, Manitoba, Canada.

John K. Marshall, Department of Medicine, McMaster University, Hamilton, Ontario, Canada.

Kumanan Wilson, Department of Medicine, University of Ottawa, Ottawa, Ontario, Canada.

Bindee Kuriya, Department of Medicine, University of Toronto, Toronto, Ontario, Canada.

J. Antonio Aviña-Zubieta, Department of Medicine, The University of British Columbia, Vancouver, British Columbia, Canada.

Paul Fortin, Department of Medicine, Université Laval, Quebec City, Quebec, Canada.
